# Biogenesis of mitochondrial β‐barrel membrane proteins

**DOI:** 10.1002/2211-5463.13905

**Published:** 2024-09-29

**Authors:** Iniyan Ganesan, Jon V. Busto, Nikolaus Pfanner, Nils Wiedemann

**Affiliations:** ^1^ Institute of Biochemistry and Molecular Biology, ZBMZ, Faculty of Medicine University of Freiburg Germany; ^2^ CIBSS Centre for Integrative Biological Signalling Studies University of Freiburg Germany; ^3^ BIOSS Centre for Biological Signalling Studies University of Freiburg Germany

**Keywords:** Mco6, Mdm10, mitochondria, outer membrane, SAM, Sam35, Sam37, Sam50, sorting and assembly machinery, β‐barrel protein

## Abstract

β‐barrel membrane proteins in the mitochondrial outer membrane are crucial for mediating the metabolite exchange between the cytosol and the mitochondrial intermembrane space. In addition, the β‐barrel membrane protein subunit Tom40 of the translocase of the outer membrane (TOM) is essential for the import of the vast majority of mitochondrial proteins encoded in the nucleus. The sorting and assembly machinery (SAM) in the outer membrane is required for the membrane insertion of mitochondrial β‐barrel proteins. The core subunit Sam50, which has been conserved from bacteria to humans, is itself a β‐barrel protein. The β‐strands of β‐barrel precursor proteins are assembled at the Sam50 lateral gate forming a Sam50‐preprotein hybrid barrel. The assembled precursor β‐barrel is finally released into the outer mitochondrial membrane by displacement of the nascent β‐barrel, termed the β‐barrel switching mechanism. SAM forms supercomplexes with TOM and forms a mitochondrial outer‐to‐inner membrane contact site with the mitochondrial contact site and cristae organizing system (MICOS) of the inner membrane. SAM shares subunits with the ER‐mitochondria encounter structure (ERMES), which forms a membrane contact site between the mitochondrial outer membrane and the endoplasmic reticulum. Therefore, β‐barrel membrane protein biogenesis is closely connected to general mitochondrial protein and lipid biogenesis and plays a central role in mitochondrial maintenance.

## Graphical abstract

Mitochondrial β‐barrel precursors are synthesized in the cytosol and imported by the translocase of the outer membrane (TOM). The sorting and assembly machinery (SAM) inserts β‐barrel membrane proteins from the intermembrane space into the outer membrane. The endosymbiotic origin of mitochondria explains the conservation of the membrane insertion mechanism and of the essential core subunit Sam50 from bacteria to humans.

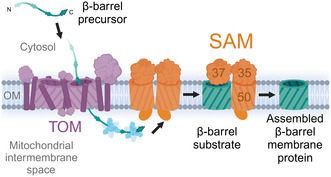

**Abbreviations**
BAM: bacterial β‐barrel assembly machinery
ER: endoplasmic reticulum
ERMES: endoplasmic reticulum‐mitochondria encounter structure
IM: inner membrane
IMS: mitochondrial intermembrane space
Mco6: Mdm10 complex protein of 6 kDa
Mdm10/12/34: mitochondrial distribution and morphology proteins
MICOS: mitochondrial contact site and cristae organizing system
MIM: mitochondrial import machinery
Mmm1: maintenance of mitochondrial morphology protein 1
MTCH2: mitochondrial carrier homolog 2
OM: outer membrane
POTRA: polypeptide‐transport‐associated domain
SAM: mitochondrial sorting and assembly machinery
SEC translocase: bacterial translocase of the secretory system
TIM: translocase of the inner mitochondrial membrane
TOM: translocase of the outer mitochondrial membrane

## β‐Barrel membrane proteins

Mitochondria are eukaryotic organelles descended from a Gram‐negative α‐proteobacterial ancestor in an ancient endosymbiotic event. Over the course of evolution, mitochondria have integrated with the eukaryotic host cell in many ways, but retained essential key features of the bacterial ancestor. These features include many metabolic enzymes such as the citric acid cycle, the respiratory chain, and the ATP synthase. Another essential ancestral feature is the presence of membrane integral β‐barrel proteins in the mitochondrial outer membrane, which are found in no other eukaryotic host cell membrane with the exception of other endosymbiosis‐derived organelles like plastids [1, 2, 3]. Almost all integral membrane proteins of the Gram‐negative bacterial outer membrane are β‐barrels with an even number of β‐strands [4]. Early in mitochondrial evolution, one of these β‐barrel proteins evolved the function of translocating proteins from the cytosol across the outer membrane into the mitochondria. This process is essential to retain mitochondrial function while genes encoding mitochondrial proteins were transferred from the mitochondrial genome to the nucleus. Presently, the vast majority (~ 99%) of mitochondrial proteins are encoded in the nucleus and translated in the cytosol. All of these precursor proteins that are imported across the outer membrane are translocated by the β‐barrel protein core subunit Tom40 of the translocase of the outer mitochondrial membrane (TOM) [5]. The second essential outer membrane β‐barrel protein is Sam50, which is required for inserting β‐barrel proteins, like Tom40 and the outer membrane metabolite channeling β‐barrel of the porin/VDAC (voltage‐dependent anion channel) family, into the outer mitochondrial membrane (Table 1). Due to the crucial nature of β‐barrel proteins, this bacterial feature was universally retained in all mitochondria and a significant proportion of the mitochondrial outer membrane proteome is still made up of β‐barrels [6, 7].

**Table 1:** β‐barrel membrane protein substrates of the mitochondrial sorting and assembly machinery (SAM).

Eukaryotic group	Substrate name	Number of β‐strands
Fungi (*S. cerevisiae*)	Sam50	16
	Tom40	19
	Por1	19
	Por2	19
	Mdm10	19
Mammals (*H. sapiens*)	SAMM50	16
	TOMM40	19
	VDAC1	19
	VDAC2	19
	VDAC3	19

**Fig. 1:**
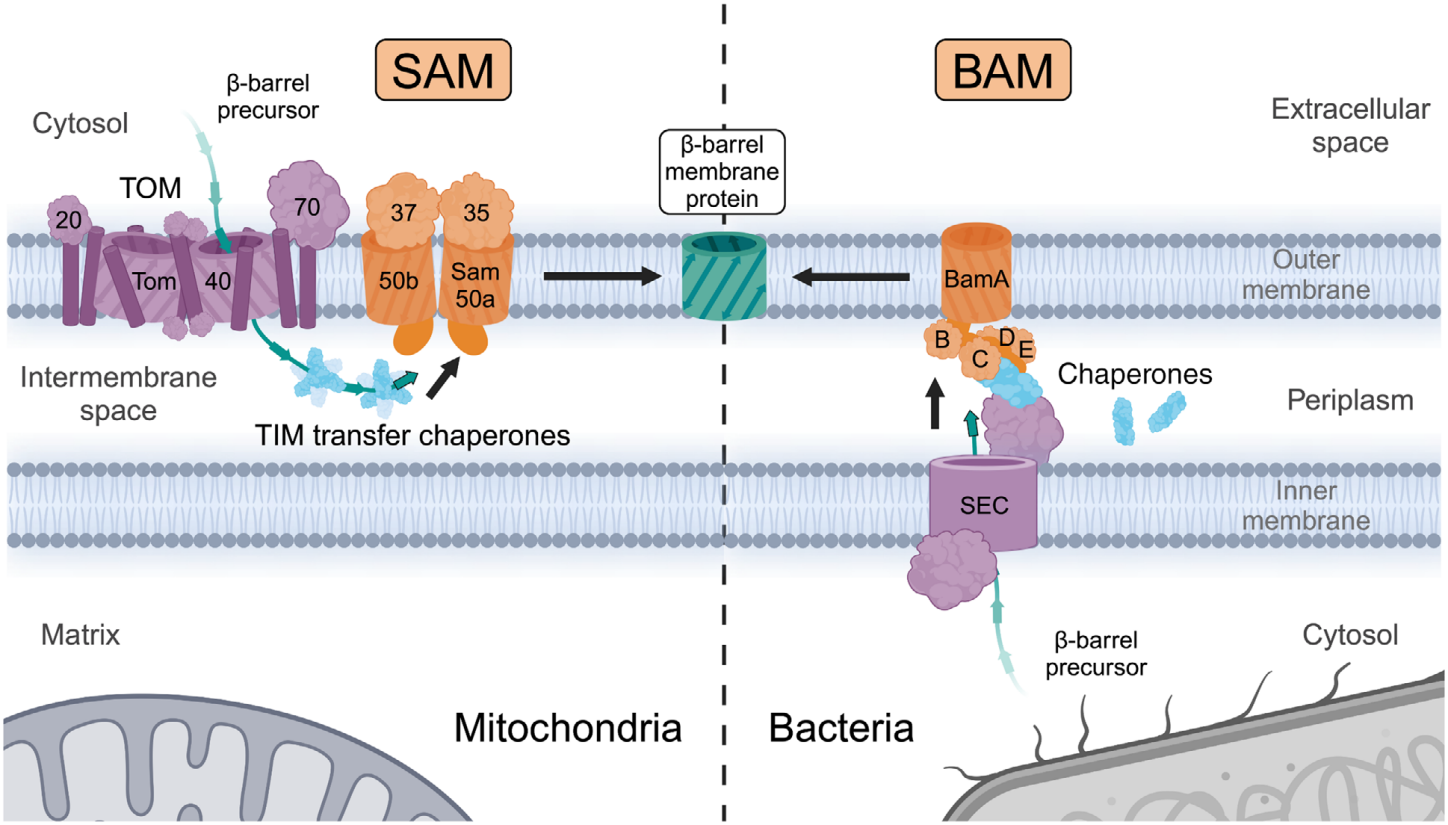
β‐barrel membrane protein biogenesis in mitochondria and Gram‐negative bacteria. Mitochondrial β‐barrels are synthesized in the eukaryotic cytosol, translocated across the outer membrane by the TOM complex, and guided by the TIM transfer chaperones across the intermembrane space to the SAM complex, where they are inserted into the outer membrane. Bacterial β‐barrels are synthesized in the cytosol, translocated across the inner membrane by the SEC translocase, chaperoned through the periplasm by a system of chaperones, and inserted into the outer membrane by the BAM complex. Although mitochondrial and bacterial β‐barrels are synthesized in opposite compartments related to the outer membrane, they are conservatively inserted into the outer membrane from the intermembrane space/periplasmic side by the homologous Omp85 family proteins Sam50 and BamA respectively. BAM, β‐barrel assembly machinery; SAM, sorting and assembly machinery; TIM, translocase of the inner mitochondrial membrane; TOM, translocase of the outer membrane.

The machineries for β‐barrel membrane protein biogenesis were discovered in mitochondria and Gram‐negative bacteria. In mitochondria, it is named the sorting and assembly machinery (SAM), or topogenesis of mitochondrial outer membrane β‐barrel proteins (TOB) complex [8, 9], and in bacteria the β‐barrel assembly machinery (BAM) [10, 11]. Similar to most mitochondrial proteins, all mitochondrial β‐barrel membrane proteins are encoded in the nucleus and translated in the cytosol. These β‐barrel precursor proteins are imported across the outer mitochondrial membrane into the intermembrane space by the TOM complex. Intermembrane space transfer chaperones guide the β‐barrel preproteins to the SAM complex, which inserts them into the outer membrane (Fig. 1) [12]. In Gram‐negative bacteria, β‐barrel membrane proteins are transported across the inner bacterial membrane by the SEC complex. Periplasmic chaperones support the transfer to the BAM complex, which inserts the β‐barrel precursor proteins into the outer bacterial membrane (Fig. 1) [13, 14, 15, 16, 17]. The core mitochondrial SAM complex subunit, Sam50, belongs to the Omp85 protein superfamily and is homologous to the bacterial core subunit BamA of the BAM complex [8, 10, 18, 19]. The conservation of the machineries for the membrane insertion of β‐barrel proteins from bacteria to mitochondria explains why mitochondrial β‐barrel proteins are translocated across the outer mitochondrial membrane before they are inserted from the intermembrane space side into the outer membrane. The SAM complexes consist of the membrane integral β‐barrel subunits Sam50 and Mdm10, the α‐helical outer membrane protein Mco6, and the peripheral cytosol‐facing subunits Sam35 and Sam37 in yeast (Table 2) [9, 20, 21, 22, 23, 24]. This review focuses predominantly on mitochondrial β‐barrel membrane protein biogenesis in yeast, as this is by far the best‐analyzed organism to date. In addition to SAMM50, animals contain two to three cytosolically exposed metaxins (MTX1, MTX2, and MTX3), which are related to Sam35 and Sam37 (Table 2) [25, 26, 27]. Recent efforts including high‐resolution structures have greatly advanced our understanding of mitochondrial β‐barrel membrane protein biogenesis at a molecular level. The roles of peripheral SAM subunits have been further elucidated and the role of Mco6 as a novel SAM subunit was recently identified. Another emerging concept of the SAM complex, in addition to its role in β‐barrel biogenesis, is its role as a hub of interactions between mitochondrial outer and inner membrane complexes.

**Table 2:** Protein components of the mitochondrial sorting and assembly machinery (SAM).

Eukaryotic group	Name	Alias	kDa
Fungi (*S. cerevisiae*)	Sam50	Tob55, Omp85	54
	Sam37	Mas37, Tob37, Tom37	37
	Sam35	Tob38, Tom38	37
	Mdm10		56
	Mco6		6
Mammals (*H. sapiens*)	SAMM50		52
	Metaxin‐1	MTX1	51^a^
	Metaxin‐2	MTX2	30^a^
	Metaxin‐3	MTX3	35^a^

^a^ Canonical sequence (Isoform 1).

## Mechanism of mitochondrial β‐barrel membrane protein biogenesis

**Fig. 2:**
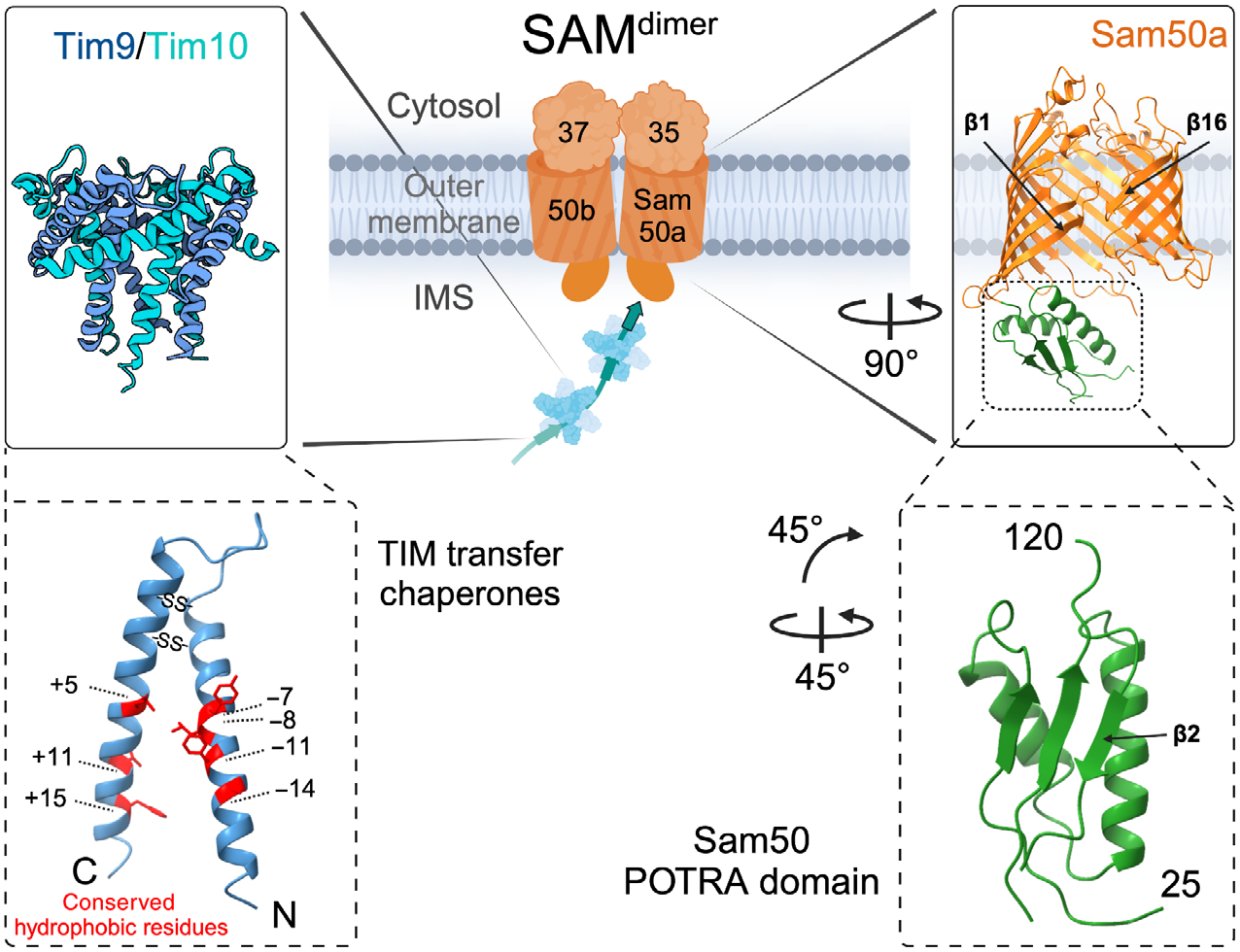
Translocase of the inner mitochondrial membrane (TIM) transfer chaperone and Sam50 POTRA domain structures. The TIM transfer chaperones Tim9/Tim10 (and Tim8/13) form an alternating hexameric structure. Each subunit contains intramolecular disulfide bonds and conserved hydrophobic residues (in positions + and ‐ with respect to the residues involved in disulfide bond formation) in their N‐ and C‐terminal helices that form a precursor binding cleft. Sam50 contains one N‐terminal POTRA domain in the intermembrane space (IMS). POTRA β‐strand 2 is available for β‐strand augmentation by the substrate and thus contributes to β‐barrel membrane protein assembly. POTRA, polypeptide‐transport‐associated domain.

Mitochondrial β‐barrel membrane proteins begin their biogenesis in the cytosol where they are post‐translationally chaperoned by the cytosolic heat shock proteins Hsp70 and Hsp40 and targeted to the TOM complex receptors Tom20 and Tom70 (Fig. 1) [28, 29, 30, 31]. The targeting signal to bind the receptors and traverse the TOM complex is a β‐hairpin composed of two adjacent antiparallel β‐strands [9, 32]. As all membrane β‐strands are amphipathic in nature, the β‐hairpin can be regarded as a structure resembling the characteristics of the classical amphipathic (α‐helical) mitochondrial presequence and as such likely binds to similar sites on the TOM receptors. The receptors are not strictly required, as artificial β‐barrel proteins can assemble in mitochondria independently of the receptors [33]. Once the β‐barrel precursor arrives at the TOM complex, it binds to residues within the Tom40 channel, which drives the translocation across the outer membrane (Fig. 1) [34]. As precursors cross the intermembrane space from TOM to SAM, TIM transfer chaperone complexes Tim9/10 and Tim8/13 prevent their aggregation [35, 36, 37]. The TIM transfer chaperones form hexameric circular complexes with alternating subunits. Each subunit contains intramolecular disulfide bonds and extended N‐ and C‐terminal α‐helices with conserved hydrophobic residues pointing into the cleft in between the helices (Fig. 2) [38, 39]. The interactions involve many loose precursor associations within the hydrophobic clefts of the TIM transfer chaperones such that precursors can be bound but also be efficiently released as they are membrane‐inserted without any external energy input [36, 40].

**Fig. 3:**
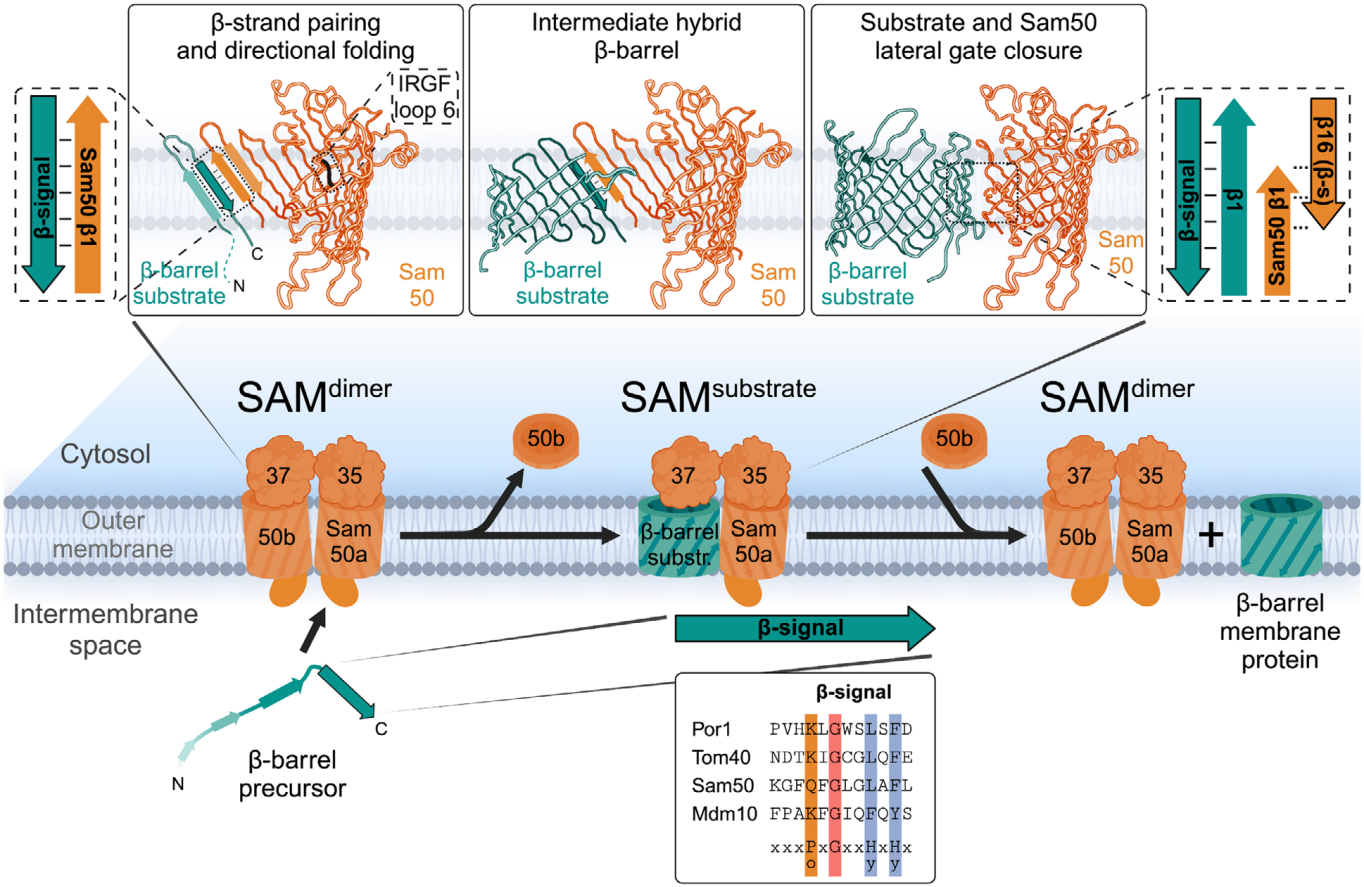
Membrane insertion of β‐barrel precursors at the SAM complex by β‐barrel switching. The last C‐terminal β‐strand of the substrate, which contains a conserved β‐signal, binds to the SAM^dimer^ complex by pairing with the first β‐strand of Sam50a. While the β‐signal remains bound to Sam50a, additional β‐strands are sequentially inserted into the membrane from C‐ to N terminus, likely as β‐hairpins. This is termed the hybrid barrel stage. The growing substrate β‐barrel displaces Sam50b. Once the substrate barrel is fully folded, its terminal β‐strands pair, replacing the β‐signal pairing with Sam50a and closing the lateral gates of both β‐barrels. The newly assembled β‐barrel precursor is released into the outer membrane and switched by Sam50b to re‐form the SAM^dimer^ complex. Hy, hydrophobic residues; Po, polar residues; SAM, sorting and assembly machinery.

The conserved β‐signal within the C‐terminal β‐strand of mitochondrial β‐barrel membrane proteins targets the precursor proteins to the SAM complex (Fig. 3, bottom) [41]. With the help of the Sam50 cytosolic loop 6 containing the conserved IRGF motif, the β‐signal binds in an antiparallel orientation to the N‐terminal β‐strand of Sam50 (Fig. 3 left panel on the top) [42]. Sam50 and the homologous bacterial BamA have 16 β‐strands each with weakly interacting terminal β‐strands (fewer inter‐strand hydrogen bonds) unlike other β‐barrel membrane proteins that do not function as β‐barrel insertases (Fig. 3 right panel on the top). The dynamics of these terminal β‐strands allows Sam50 to readily bind the β‐signal of incoming β‐barrel precursors. Both open and closed states of Sam50/BamA have been observed in cryo‐EM structures, by intramolecular crosslinking and molecular dynamics simulations [42, 43, 44, 45, 46]. The next step after SAM binding is insertion of the precursor into the membrane. Both Sam50 and BamA are hydrophobically mismatched with the membrane near their terminal β‐strands and therefore locally thin/distort the membrane to facilitate protein insertion [47, 48, 49, 50, 51]. This is a common strategy by membrane insertases to make the thermodynamics of insertion more favorable [52]. It is unclear how many β‐strands are inserted into the membrane at once, but it seems likely that β‐hairpins are inserted into the membrane sequentially from C‐ to N terminus [42, 53, 54]. Therefore, the hydrophilic loops of each β‐hairpin are likely shielded from the membrane by the interior of the Sam50 barrel and loop 6 as the β‐hairpin is inserted.

**Fig. 4:**
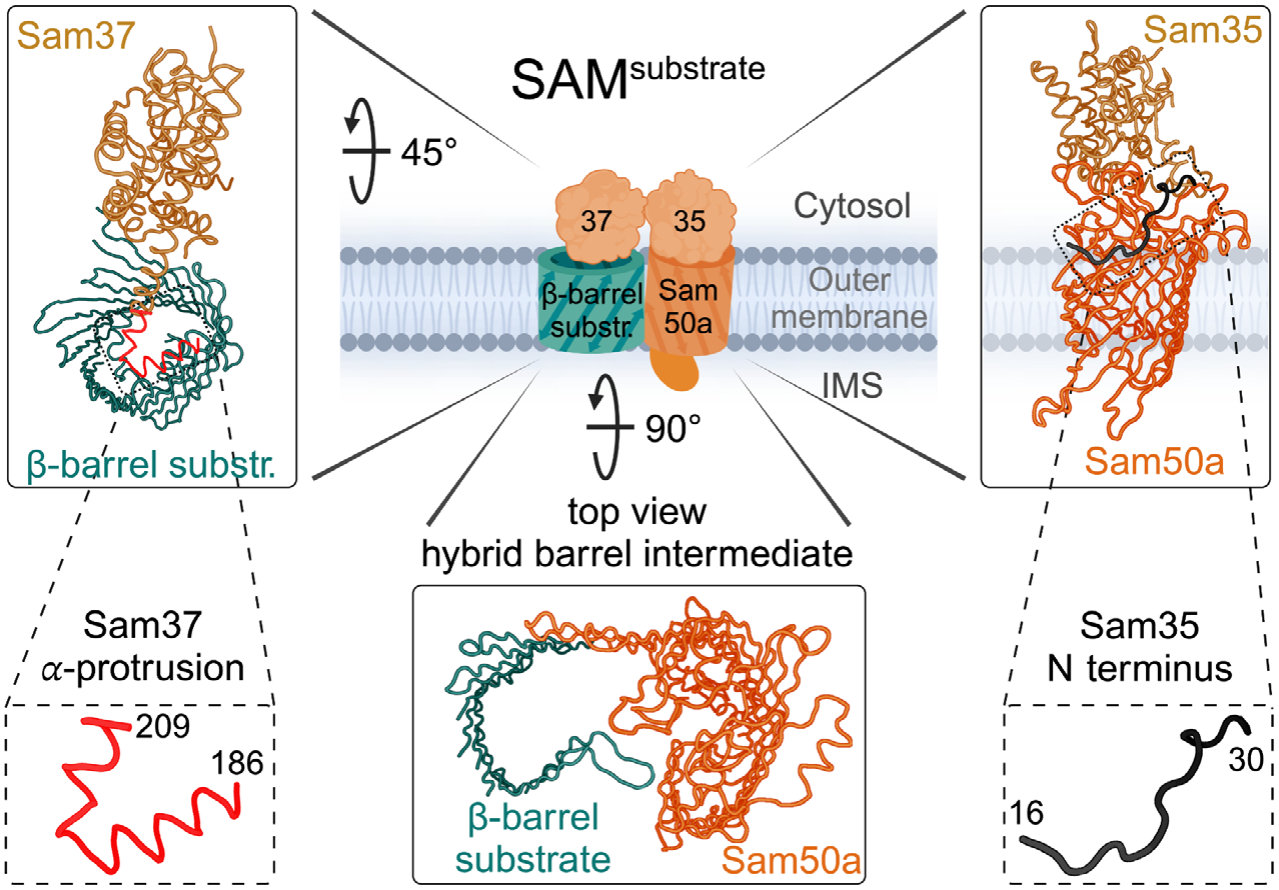
Role of peripheral cytosolic subunits Sam35 and Sam37. Sam35 sits atop Sam50a, interacting with the barrel wall with its N terminus. Sam35 assists in substrate binding to Sam50a. Sam37 is similar to Sam35 in structure and its ability to bind mitochondrial β‐barrels from the cytosolic side. Sam37 contains an α‐protrusion which is able to bind into the lumen of the growing β‐barrel substrate, thereby stabilizing the β‐barrel during its formation.

During insertion of sequential β‐hairpins, a hybrid barrel is formed in the plane of the outer membrane between the β‐barrel precursor and Sam50 with a strong interaction (hydrogen‐bond pairing) between the Sam50 N‐terminal β‐strand with the C‐terminal precursor β‐strand (Figs 3 and 4, middle panels) [42, 54]. In contrast, the C‐terminal β‐strands of Sam50 and the assembling N‐terminal β‐strands of the substrate curve similar to the mature barrel structures, such that they associate loosely with each other (without hydrogen‐bond pairing) (Figs 3 and 4, middle panels) [42, 54]. The hybrid barrel model was initially suggested in the bacterial field based on the observation that BamA lateral gate opening is important for its function [45] and that a folded passenger domain of an autotransporter secreted in *Escherichia coli* requires the combined hybrid barrels of the autotransporter β‐barrel domain and the BamA barrel to accommodate translocation of the folded domain [55, 56]. The model was proven in mitochondria by extensive crosslinking experiments [42] and recently validated by the Tom40 precursor‐SAM structure for mitochondria [54] and precursor‐BAM structures for bacteria [57, 58].

It is feasible that the formation of the hydrogen bonds between the first and the last β‐strand of the substrate and reformation of the lateral gate of Sam50 is sufficient to disrupt the hybrid barrel interaction between the substrate and Sam50, such that β‐barrel biogenesis can proceed without external energy input [59].

## Eukaryotic specific subunits for β‐barrel membrane protein biogenesis

Even though the β‐barrel membrane insertion machineries SAM in mitochondria and BAM in bacteria operate by a similar core mechanism due to the conserved subunits, Sam50 and BamA, there are also fundamental differences. The additional mitochondrial SAM complex subunits are either peripheral subunits on the cytosolic face of the SAM complex (Sam35 and Sam37) or integral outer membrane proteins (Mdm10 and Mco6) and have no homology to bacterial BAM subunits. In contrast, all additional bacterial BAM subunits (BamB to BamE) are periplasmic proteins peripherally associated together with BamA [13, 14, 16, 17]. It is tempting to speculate that the BAM machinery evolved to operate without extracellular proteins to protect the BAM machinery from the extracellular environment, while the mitochondrial machinery could evolve with cytosolic subunits on the opposing face of the outer membrane and with an α‐helical outer membrane protein, since these are common in the mitochondrial outer membrane (Fig. 1).

Sam50 and BamA contain polypeptide‐transport‐associated (POTRA) domains located in the intermembrane space or periplasm respectively. POTRA domains adopt a three‐stranded β‐sheet structure overlaid with a pair of antiparallel helices with β‐α‐α‐β‐β arrangement (Fig. 2 right) [60, 61]. The POTRA domain β‐strand 2 is available for β‐strand augmentation by substrate proteins, and thus, the POTRA domains were suggested to be crucial for targeting the β‐barrel substrates to SAM and BAM [62, 63]. Even though numerous POTRA domains can be deleted in the bacterial system, the presence of a single POTRA domain is essential for BamA function [64]. In contrast, the single POTRA domain of mitochondrial Sam50 is not essential, but likely supports β‐barrel protein assembly and substrate release [41, 65].

The two additional SAM complex core subunits Sam35 and Sam37 are structurally similar peripheral membrane proteins that cap different mitochondrial β‐barrel membrane proteins (Sam50, Mdm10, or growing substrate barrels). Sam35 was discovered shortly after Sam50, but Sam37 was identified with a proposed function as a mitochondrial import receptor long before this [66]. These peripheral subunits do not have strong sequence conservation across species, but are generally present throughout. In some species, Sam37 even has a transmembrane segment that makes it an integral outer membrane protein [43]. Sam35 and Sam37 have a glutathione‐S‐transferase (GST)‐like fold and are related to metaxins (MTX1, MTX2, and MTX3) in humans that are also known to be involved in β‐barrel biogenesis together with human SAMM50 (Table 2) [25, 27, 43].

Sam35 (Tom38, Tob38) is the other essential mitochondrial SAM subunit and caps the β‐barrel protein Sam50 from the cytosolic face [21, 23, 24, 43, 46, 54, 67]. The N terminus of Sam35 is essential and associates with the cytosolic rim at the C terminus of the Sam50 barrel (Fig. 4, right) [46]. This suggests that Sam35 is involved in Sam50 lateral gate opening, which is consistent with the crucial role of Sam35 for β‐signal binding at the SAM complex [41, 68]. Although bacteria do not have a Sam35 homolog, the BAM complex contains a second essential subunit BamD associated on the opposing periplasmic side. However, BamD does not play a catalytic role in outer membrane protein assembly, but rather functions to regulate the activity of BamA by substrate binding [69, 70].

**Fig. 5:**
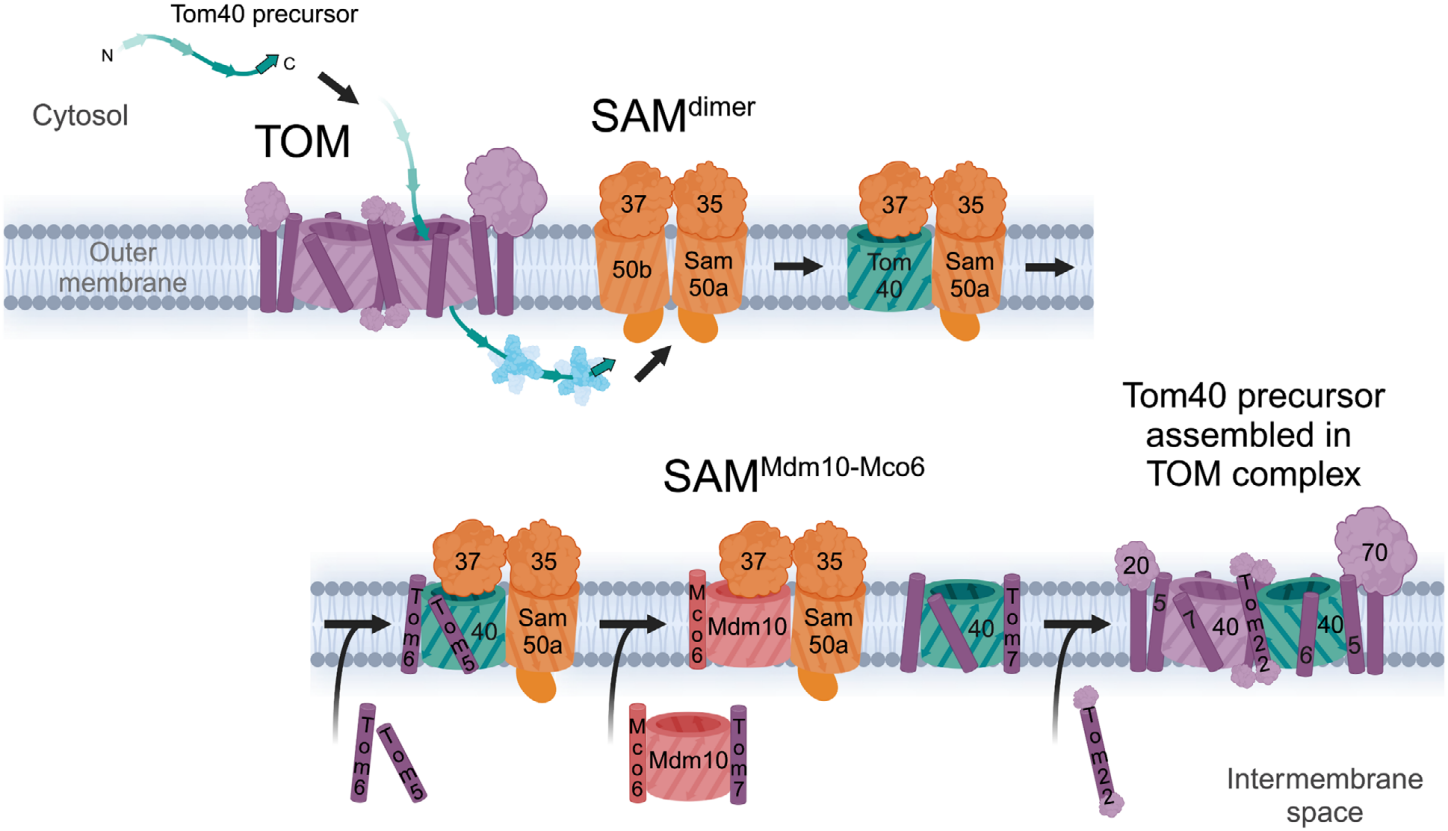
Tom40 assembly at SAM. Tom40 follows the general β‐barrel insertion pathway in terms of binding to Sam50a via a β‐signal and forming a hybrid barrel structure (upper panel). However, unlike other β‐barrels, Tom40 assembles with small α‐helical transmembrane proteins while bound to the SAM complex (lower panel). Tom5 and Tom6 assemble with the Tom40 precursor while still bound to SAM. Release of Tom40 requires another β‐barrel protein, Mdm10, which displaces the Tom40 substrate at SAM via interactions with Sam50a and Sam37. During the displacement process, Mdm10 delivers another small translocase of the outer membrane (TOM) protein, Tom7, to Tom40‐5‐6. Mdm10 is itself stabilized by an additional small membrane protein, Mco6. The final core TOM subunit, Tom22, is assembled with Tom40‐5‐6‐7, which allows for dimerization and formation of the mature TOM complex.

Mitochondrial Sam37 (Mas37, Tom37, and Tob37) is not essential for cell viability but it is crucial for efficient β‐barrel protein biogenesis [9]. Sam37 is found in three different SAM complex variants: (a) the SAM^dimer^ complex with two copies of Sam50 (named Sam50a and Sam50b) associated with their lateral gates facing each other (Fig. 3) [46] (b) the SAM^Mdm10^ complex with the β‐barrel proteins Sam50a and Mdm10 (Fig. 5) [46] (c) the SAM^substrate^ complex with Sam50a and the β‐barrel protein substrate (Figs 3, 4, 5) [54, 67]. Sam37 caps the dynamically associated β‐barrel component of each of these complexes from the cytosolic side of the outer membrane.

Upon engagement of the β‐barrel substrate with the SAM complex, the substrate β‐signal binds to the N‐terminal β‐strand of Sam50a and thereby displaces Sam50b. This mechanism of SAM^dimer^ to SAM^substrate^ complex conversion for mitochondrial β‐barrel biogenesis was termed β‐barrel switching (Fig. 3) [46]. Sam37 stabilizes the growing barrel of the substrate from the cytosolic side and contains an α‐helical protrusion that can insert into the substrate β‐barrel lumen (Fig. 4, left). Thereby, Sam37 and its α‐helical protrusion enhance the efficiency of mitochondrial β‐barrel membrane protein biogenesis and substrate release [9, 54, 68]. Thus, Sam37 is required for the efficient barrel closure of the substrate and functions as the molecular cooper of the SAM complex.

In contrast to bacterial β‐barrel proteins with an even number of β‐strands, the major mitochondrial β‐barrel proteins of the porin/VDAC and Tom40 families contain 19 β‐strands with an α‐helical N terminus, which can fold into the lumen of the barrel (Table 1) [71, 72, 73, 74]. These α‐helical N termini of the β‐barrel substrates are crucial for barrel formation and efficient substrate release from the SAM complex [54]. The lipid composition of the mitochondrial outer membrane also greatly contributes to an efficient assembly of β‐barrel membrane proteins. Cardiolipin, phosphatidylcholine, or phosphatidylethanolamine depletion greatly reduces the biogenesis of β‐barrel proteins [75, 76, 77].

Taken together, Sam35 is essential for the engagement of the β‐barrel protein substrate with Sam50. Sam37, the α‐helical N‐termini of mitochondrial β‐barrel proteins, and the physical properties of the lipid bilayer are crucial for the formation of β‐barrels in the mitochondrial outer membrane.

## Assembly of the TOM complex

The main mitochondrial entry gate for precursor proteins in the outer membrane, the TOM complex, consists of the β‐barrel protein Tom40 and six α‐helical membrane proteins: the receptors Tom20, Tom22, and Tom70, and the small proteins Tom5, Tom6, and Tom7 (Fig. 5). The structurally best‐characterized stoichiometry of the TOM core complex consists of two copies of the β‐barrel protein Tom40 and two copies each of the α‐helical subunits Tom5, Tom6, Tom7, and Tom22. The two Tom40 β‐barrels are tethered by the α‐helical transmembrane segments of the Tom22 proteins, while the small α‐helical Tom proteins Tom5, Tom6, and Tom7 specifically associate individually with the Tom40 barrels [71, 74, 78, 79, 80].

Different assembly intermediate structures of Tom40 in the SAM^substrate^ complex were obtained. In the hybrid barrel intermediate structure with 12 membrane‐inserted β‐strands, the N‐terminal β‐strand 1 of Tom40 is found in the lumen of the growing barrel [54]. The Sam37 α‐helical protrusion, which dynamically projects into the substrate β‐barrel, assists in assembling Tom40 (Fig. 4) [9, 54]. After all the other β‐strands are inserted into the membrane, the N‐terminal α‐helical domain of Tom40 displaces β‐strand 1 from the Tom40 lumen allowing it to hydrogen bond with the last β‐strand, closing the barrel and completing the folding process. The small TOM proteins, Tom5 and Tom6, assemble with Tom40 before release from the SAM complex [81, 82]. It can be speculated that the association of Tom5 stabilizes the Tom40 β‐barrel domain and thus increases the efficiency of Tom40 β‐barrel formation at the SAM complex. In the SAM^substrate^ structures with a completely folded β‐barrel of Tom40, the structure of Tom40 already resembles its conformation in the mature TOM complex [67, 71, 74]. Moreover, in the SAM^Tom40‐5‐6^ structure, Tom5 and Tom6 are associated with Tom40 like in the assembled TOM complex [67, 71, 74]. The isolation of the stable SAM^Tom40‐5‐6^ intermediate indicates that folding of the β‐barrel and release from SAM are distinct steps for Tom40 [67].

The release of the Tom40‐Tom5‐Tom6 intermediate from the SAM complex is greatly enhanced by another outer membrane β‐barrel protein called Mdm10 (mitochondrial distribution and morphology protein 10) together with the α‐helical outer membrane protein Mco6 (Mdm10 complex protein of 6 kDa) (Fig. 5) [20, 22, 83]. Mco6 forms a complex with Mdm10, which is related to Tom40, and is thought to stabilize the β‐barrel protein [20, 84]. The Mdm10‐Mco6 complex displaces the Tom40‐Tom5‐Tom6 intermediate from the SAM complex by β‐barrel switching and the SAM^Mdm10‐Mco6^ complex is formed [20, 46, 83]. The Mdm10‐Mco6 interaction with SAM represents a subpopulation of the SAM complexes [20]. After the release of the Tom40‐Tom5‐Tom6 intermediate from SAM, it assembles with Tom7 and with Tom22. Mdm10 binds Tom7 on the opposite side of its β‐barrel domain with respect to Mco6; however, in the SAM^Mdm10‐Mco6^ complex, the binding site for Tom7 is occupied by Sam50 [20]. Therefore, it seems likely that Tom7 is directly transferred from Mdm10 to Tom40 concomitantly during the β‐barrel switching process (Fig. 5) [85, 86]. Mdm10 and Mco6 are likely also involved in the assembly of Tom22 with Tom40, since *mdm10* and *mco6* mutants display a reduced assembly of Tom22 and, in addition, a diminished protein level of Tom22 [20, 22]. One can speculate that a Tom7‐Mdm10‐Mco6 complex is specifically required for the release of the Tom40‐Tom5‐Tom6 intermediate to coordinate the transfer of the phospholipid which binds to the Tom40 lateral gate with the assembly of Tom7 and Tom22 [71]. The SAM^Mdm10‐Mco6^ complex is likely in an equilibrium with other SAM complexes, ERMES, and β‐barrel substrates.

In summary, the β‐barrel switching mechanism for the biogenesis of the TOM complex involves the displacement of Sam50b from the SAM^dimer^ complex by the Tom40 precursor (Fig. 3) and the reverse displacement of the Tom40‐Tom5‐Tom6 precursor intermediate by Mdm10‐Mco6 (Fig. 5).

## Interactions of the SAM complex

**Fig. 6:**
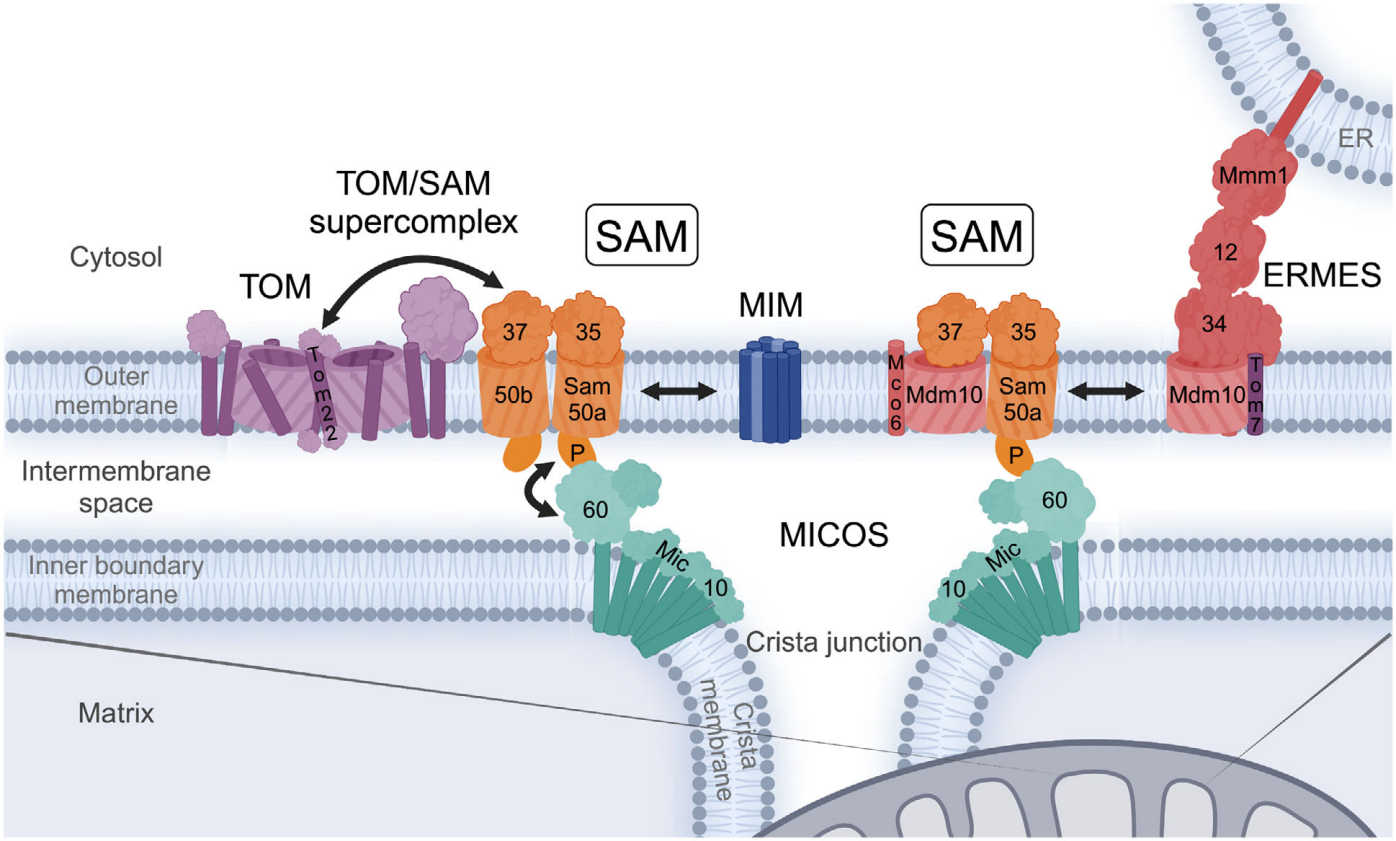
Interactions of the sorting and assembly machinery (SAM) complex with other mitochondrial protein machineries. SAM forms a supercomplex with translocase of the outer membrane (TOM) through interactions of Sam37 and Tom22. This couples β‐barrel substrate translocation across and insertion into the outer membrane. SAM interaction with the α‐helical outer membrane insertase mitochondrial import machinery (MIM) facilitates small TOM subunit assembly with Tom40 at the SAM complex. Mdm10 is a subunit of both SAM and ERMES to regulate mitochondrial protein and lipid biogenesis. The Sam50 POTRA domain interacts with the MICOS complex at cristae junctions in the inner membrane and forms a mitochondrial contact site between the outer and the inner mitochondrial membranes. All of these interactions place SAM in a central position with regard to protein and lipid biogenesis in mitochondria. ERMES, endoplasmic reticulum‐mitochondria encounter structure; MICOS, mitochondrial contact site and cristae organizing system.

The SAM complex interacts with several other protein complexes of mitochondria (Fig. 6), but the most important interaction for β‐barrel biogenesis is that with the TOM complex. The TOM‐SAM supercomplex is formed by interactions between the cytosolic domains of Tom22 and Sam37 (Fig. 6) [87, 88]. Although not strictly essential, the formation of a TOM‐SAM supercomplex allows for the efficient coupling of β‐barrel translocation steps from translocation across the outer membrane via TOM—to insertion into the membrane via SAM. The TIM transfer chaperones do not seem to directly interact with SAM but merely prevent the aggregation of hydrophobic precursor segments as they cross the aqueous intermembrane space. Novel SAM complex/subunit interactions continue to be identified, such as the recent finding that the metaxins in human cells also link mitochondria with microtubule motor assemblies [89].

The SAM complex also interacts with the mitochondrial import complex (MIM), which facilitates insertion of α‐helical proteins into the outer membrane in yeast (Fig. 6) [90, 91, 92]. MIM consists of the two α‐helical outer membrane proteins Mim1 and Mim2, which are thought to form a channel for the insertion of α‐helical outer membrane protein substrates [93, 94, 95, 96]. SAM interacts with MIM in order to coordinate the assembly of α‐helical and β‐barrel membrane proteins [65, 90, 94]. Specifically, the interaction couples Tom5 and Tom6 insertion via MIM with the biogenesis of Tom40 at SAM (Fig. 5). Moreover, SAM subunits are also required for α‐helical protein insertion of the TOM core subunit Tom22 and the small TOM proteins Tom5, Tom6, and Tom7 [97].

In addition to these functional interactions for coupling precursor protein import (TOM) or α‐helical protein assembly (MIM), SAM also interacts with the mitochondrial contact site and cristae organizing system (MICOS) in the inner membrane forming the mitochondrial intermembrane space bridging complex (MIB) [98, 99, 100, 101, 102]. This mitochondrial membrane contact site is formed between the POTRA domain of the outer membrane protein Sam50 and the intermembrane space domain of the inner membrane protein Mic60 (Fcj1, Mitofilin) of the MICOS complex (Fig. 6) [103, 104]. Depletion of Mic60 impairs the assembly of β‐barrel membrane proteins [103]. The homo‐oligomeric MICOS core subunit Mic10 is thought to bend the inner membrane via two transmembrane segments connected by a short charged loop in the matrix to form V‐shaped structures that induce the inner membrane curvature to form crista junctions [105, 106]. The diameter of the crista junction is likely fixed by an antiparallel tetrameric assembly of the Mic60 intermembrane space domain, which connects the opposing sides of the crista junction membrane [107].

Even though the SAM complex does not directly interact with the endoplasmic reticulum‐mitochondria encounter structure (ERMES), it shares a subunit, Mdm10, that binds to either complex (Fig. 6) [108]. ERMES is responsible for physically connecting mitochondria and the ER and for transferring lipids between them [109, 110, 111]. ERMES consists of an α‐helical ER membrane protein named Mmm1 (maintenance of mitochondrial morphology 1) and two cytosolic proteins Mdm12 and Mdm34. All three ERMES proteins contain a synaptotagmin‐like mitochondrial lipid‐binding protein (SMP) domain, which form a conduit of hydrophobic cavities for the transfer of lipids between the organelles [112, 113]. The β‐barrel protein Mdm10 anchors ERMES at the mitochondrial outer membrane. Mdm10 is in an equilibrium between the SAM and ERMES complexes and Tom7 can only bind to free Mdm10 or Mdm10 within the ERMES complex, but not in the SAM^Mdm10‐Mco6^ complex [20, 85, 86]. Interestingly, the Mco6 homolog Emr1 of fission yeast (*Schizosaccharomyces pombe*) was found to regulate the number of ERMES foci, consistent with the role of Mco6 to stabilize Mdm10 [20, 114]. Tom7 overexpression increases the abundance of SAM complexes lacking Mdm10 and concomitantly enhances the β‐barrel assembly of porin [86]. This is consistent with the observation that precursors do not bind to the SAM^Mdm10‐Mco6^ complex since Mdm10 overexpression blocks substrate binding [83]. Conversely, *TOM7* deletion reduces the assembly of porin but enhances the efficiency of Tom40 assembly due to the increased availability of the assembly factor Mdm10 [85, 86]. This intricate mechanism of balancing the activities of SAM and ERMES by the TOM subunit Tom7 likely regulates the protein and lipid biogenesis of the mitochondrial outer membrane in relation to the assembled TOM complex.

All interactions considered, SAM is a central complex in mitochondrial protein and lipid biogenesis pathways through its main role as insertase for β‐barrel membrane proteins, its cooperation with the biogenesis of α‐helical outer membrane proteins (MIM), its connection to the general import pore (TOM), the sharing of the Mdm10 subunit and its regulation by Tom7 with the ER‐mitochondria lipid exchange contact site (ERMES), and its structural role for establishing mitochondrial outer‐to‐inner membrane contact sites with MICOS. It remains to be determined whether the MICOS interaction evolved simply to tether the two mitochondrial membranes or whether the close proximity of SAM and MICOS provides additional functional advantages.

## Conclusion and open questions

Ongoing interest in β‐barrel membrane protein biogenesis has produced a large step forward in recent years in our understanding of the basic mechanisms from both the mitochondrial and the bacterial fields. Before the β‐barrel assembly machineries were discovered in mitochondria (SAM) and bacteria (BAM), it had been assumed that β‐barrel membrane proteins preform in the cytosol/periplasm to insert directly into the outer membrane [115, 116]. It is now clear that the biogenesis of native β‐barrel membrane proteins requires the essential Sam50/BamA, which form 16‐stranded β‐barrels with a dynamic lateral gate in their respective outer membranes. The opening of the lateral gate is crucial so that the C‐terminal β‐signal‐strand of the precursors can bind to the N‐terminal β‐strand of Sam50/BamA to start the formation of a hybrid barrel. After formation of the hydrogen bonds between the first and the last β‐strands of the substrate, the folded β‐barrel can be released into the outer membrane. This process has been well studied in yeast and also in bacteria.

However, there are many open questions, especially for the assembly of β‐barrel proteins in metazoan/human mitochondria. How do the metaxin proteins support the β‐barrel membrane protein biogenesis compared with yeast Sam35 and Sam37 even though they are not stably associated with SAMM50? How is the TOM complex assembled in metazoan/human mitochondria? In humans, a mitochondrial carrier homolog 2 (MTCH2) was identified as a crucial protein required for the insertion of α‐helical outer membrane proteins [117]. Does MTCH2 also functionally and physically interact with SAM? How is the biogenesis of β‐barrel membrane proteins regulated under different metabolic and growth conditions?

Taken together, it is good to see that the mitochondrial and the bacterial fields managed to agree on a general mechanism for β‐barrel membrane protein biogenesis consistent with the finding that mitochondrial β‐barrel membrane proteins can be assembled in bacteria and vice versa [118, 119, 120]. Yet, much work remains to understand the intricate mechanisms for the biogenesis of this fascinating class of essential membrane proteins.

## Conflict of interest

The authors declare no conflict of interest.

## Author contributions

IG, JVB, NP, and NW conceived the manuscript and conceptualized the figures and tables. IG wrote the manuscript draft and JVB created the draft of the images and tables. All authors wrote the manuscript.
